# Viability and Radiosensitivity of Human Tumor Cells from Breast and Colon Are Influenced by *Hypericum perforatum* Extract HP01

**DOI:** 10.3390/ijms26020622

**Published:** 2025-01-13

**Authors:** Linda Rebecca Haake, Ahmed El Menuawy, Hannes Rennau, Frank Marthe, Urs Hähnel, Felix Bock, Guido Hildebrandt, Katrin Manda

**Affiliations:** 1 Department of Radiotherapy and Radiation Oncology, University Medical Center Rostock, Suedring 75, 18059 Rostock, Germany; linda.haake2@uni-rostock.de (L.R.H.); hannes.rennau@uni-rostock.de (H.R.); felix.bock@med.uni-rostock.de (F.B.); guido.hildebrandt@med.uni-rostock.de (G.H.); 2 Institute for Breeding Research on Horticultural Crops, Julius Kühn Institute (JKI), Federal Research Centre for Cultivated Plants, Erwin-Baur-Strasse 27, 06484 Quedlinburg, Germany; ahmed.menuawy@julius-kuehn.de (A.E.M.); frank.marthe@julius-kuehn.de (F.M.);

**Keywords:** *Hypericum*, St. John’s Wort, MCF-7, HT-29, ionizing radiation, clonogenic survival

## Abstract

To enhance the treatment of tumors that are resistant to radio- and chemotherapy while minimizing the side effects of radiochemotherapy, researchers are continuously seeking new active compounds for use in combination with radiotherapy. Therefore, the aim of our study was to examine the cytotoxic and radiosensitizing effects of an extract from St. John’s Wort (*Hypericum perforatum)*, referred to as HP01, on human epithelial tumor cells in vitro. The growth of MCF-7 (breast carcinoma) and HT-29 (colon carcinoma) cells was examined under the influence of HP01. In combination with radiation, the effects of HP01 on cytotoxicity and long-term survival were assessed using a colony formation assay. The number of DNA double-strand breaks was analyzed using the γH2AX assay, while cell cycle distribution was examined via flow cytometry. A growth-inhibiting and cytotoxic effect was observed for both tumor cell lines starting at a concentration of 10 µg/mL HP01. Treatment with HP01 resulted in an inhibition of clonogenic survival of tumor cells after ionizing radiation (6 Gy). The number of DNA double-strand breaks (DSBs) in tumor cells increased with HP01 treatment, but the repair of radiation-induced DNA DSBs was not affected. Cell cycle analysis revealed that HP01, in addition to radiation, enhanced G2/M arrest in MCF-7 and HT-29 cells. Overall, HP01 not only showed a growth-inhibiting effect but also demonstrated a radiosensitizing effect on human tumor cells for the first time. We conclude that the HP01-induced G2/M accumulation of cells may be the main rationale for the drug-induced radiosensitivity. It is therefore a promising candidate for combined therapy in tumor diseases and warrants further investigation.

## 1. Introduction

Alongside surgery and chemotherapy, radiotherapy represents one of the central pillars of cancer treatment. Ionizing radiation has been well established in the treatment of cancer patients for decades and remains a highly effective method for eliminating tumors [1,2]. Today, more than 50% of all cancer patients undergo radiation therapy at some point during their treatment [3].

The combination of radiation therapy and active substances has significantly improved the treatment of various cancers over the past two decades. Numerous randomized studies have demonstrated that simultaneous application of radiation and chemotherapy enhances tumor control and improves patient survival rates [4,5]. In clinical practice, several cytostatic agents, such as taxanes, cisplatin, temozolomide, and 5-fluorouracil, have proven beneficial in combination with radiation therapy [6,7,8].

However, the high radiation doses required to effectively kill tumors often result in significant side effects that impact the quality of life for cancer patients. Therefore, there is an urgent need to focus on methods to reduce the radiation dose while maintaining effectiveness. One possibility is to identify new natural substances that can enhance the effect of radiation and, in turn, reduce the required radiation dose. Natural substances often produce fewer side effects compared to chemically synthesized drugs. The aim of this work was to explore the combined effects of an extract from St. John’s Wort (*Hypericum perforatum*) and ionizing radiation on human tumor cell lines.

Given their diverse pharmacological characteristics, *Hypericum* species and their constituents are of great interest to modern medicine. *Hypericum perforatum* is a perennial herb native to Europe, Asia and North Africa. It contains numerous bioactive compounds such as hypericin, hyperforin, hyperoside, and skyrin, as well as flavonoids such as flavones, flavonol, and tannins [9,10]. These bioactive compounds are well known for their pharmacological characteristics and can be extracted using various methods such as ethyl acetate, water, and ethanol extraction. The composition of St. John’s Wort varies depending on the species. But even within a species, the proportions of specific bioactive components depend on the breeding method. Given the different functions of these compounds, drawing generalized conclusions about the effects of St. John’s Wort is challenging. Therefore, our study precisely determined the contents of hypericin, hyperforin, rutin, and flavonoids.

*Hypericum* extracts are used to treat a range of conditions, including fibrosis and neuralgia, and serve as alternatives to conventional antidepressants [11]. A key bioactive compound in *Hypericum* extracts is hyperforin, known for its anti-inflammatory, antitumor, and antibacterial features [12,13]. In dermatology, St. John’s Wort is utilized in creams to treat mild atopic dermatitis [14]. Furthermore, hypericin is used as a photosensitizer in photodynamic therapies [15].

Interactions between *Hypericum* extracts and cancer medications have also been observed. *Hypericum* extracts have been shown to down-regulate the expression of multidrug resistance-protein 1 (MDR-1) mRNA and inhibit MDR1-mediated transport, both of which are important mechanisms of multidrug resistance responsible for the failure of various chemotherapies [16]. Overall, the various applications of *Hypericum* extracts demonstrate the broad therapeutic potential of this plant in medicine.

Initial studies investigating the effects of *Hypericum* extracts and their bioactive ingredients in oncology have already been conducted. Several in vitro studies have focused on the effects of *Hypericum* extracts and the bioactive component hypericin on human tumor cells. Treatment with 0.1 mg/mL–0.5 mg/mL *Hypericum triquetrifolium* extract (HTE) did not reduce the viability of colon cancer cells (HCT-116) [17]. However, HTE concentrations above 0.5 mg/mL caused significant lactate dehydrogenase (LDH) release, indicating cytotoxicity. Treatment of A549 lung cancer cells with hyperoside (10 µM, 50 µM, and 100 µM) for 12 h, 24 h and 48 h resulted in a time- and concentration-dependent inhibition of cell proliferation [18]. Authors described increased protein expression of caspase-3, an apoptosis effector activated by caspase-9 and caspase-8, leading to the coordinated dismantling of cell structures during apoptosis. Another study showed that *Hypericum* extracts could reduce the growth of K562 cells and induce different degrees and kinetics of apoptosis depending on the species and method of breeding used [19]. The authors highlighted the efficacy of the different bioactive compounds in *Hypericum perforatum*. Cytotoxicity and growth inhibition have also been described in lymphoma, leukemia, and glioblastoma cells [20,21], as well as in cell carcinoma of the bladder [22]. Overall, extracts and bioactive components of *Hypericum* exhibited antiproliferative effects on human tumor cells. This was accompanied by increased expression of caspase-3 protein, which indicates induced apoptosis. Further studies are necessary to investigate the effects of bioactive compounds of *Hypericum* on tumor cells and to develop potential applications in cancer therapy.

As previously mentioned, since 50% of all tumor patients receive radiotherapy, the influence of active substances on the radiation response of tumor cells is of great importance. Therefore, the aim of our study was to determine whether a natural extract (HP01) from St. John’s Wort (*Hypericum perforatum*) can have any radiation-sensitizing effect on tumor cells at all. We chose two human tumor cell lines that represent highly prevalent tumor entities: MCF-7 (breast carcinoma) and HT-29 (colon carcinoma).

Breast carcinoma is the most common cancer among women worldwide; in 2022, there were 2.3 million diagnoses and 670,000 deaths globally. In countries with a very high Human Development Index, one in 12 women will be diagnosed with breast cancer in their lifetime, leading to death in 1 of 71 cases [23]. Colorectal carcinoma is the third most commonly diagnosed cancer globally and the second leading cause of cancer-related deaths in Europe [24]. Radiotherapy is an established treatment method for both types of tumors. The use of *Hypericum* could provide new ways to reduce radiation doses while enhancing the effectiveness of treatment.

## 2. Results

### 2.1. Growth of Tumor Cells Influenced by Hypericum perforatum Extract HP01

**Figure 1:**
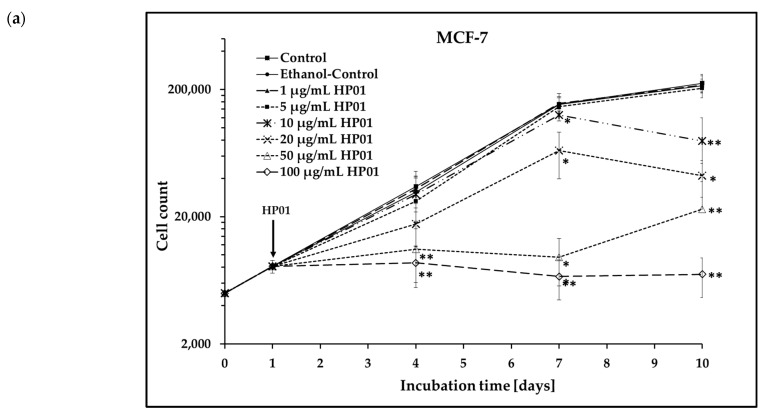

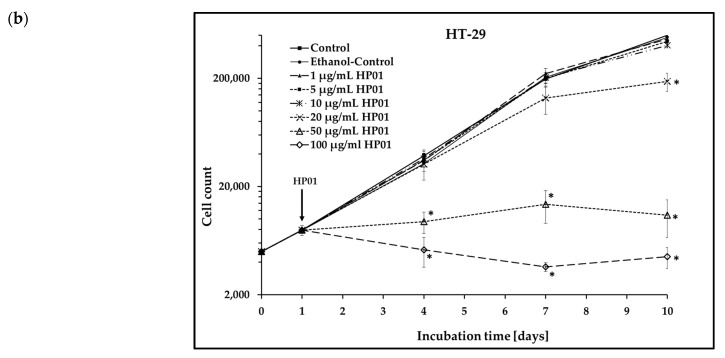
Growth of tumor cells treated with *Hypericum perforatum* extract HP01. The cells were seeded at day 0. HP01 was added to the cells at day 1 of incubation after a 24 h cell adhesion period to (**a**) MCF-7 cells and (**b**) HT-29 cells. Error bars represent the standard deviations of six (MCF-7: 1, 10, 50, 100 µg/mL HP01), three (MCF-7: 5, 20 µg/mL HP01), and four (HT-29) separate experiments; wells were assayed in triplicate in each of the different experiments. Significance was calculated for each day’s approaches (control versus treated sample). Asterisks illustrate significances: * *p* ≤ 0.05, ** *p* ≤ 0.01.

Growth curves of the MCF-7 and HT-29 cells were generated to analyze the impact of HP01 on cell growth. Cells were treated with various concentrations of HP01 and incubated for up to 9 days (Figure 1).

As shown in Figure 1a, HP01 concentrations of 1 µg/mL and 5 µg/mL had no effect on the MCF-7 cells. Starting at a concentration of 10 µg/mL HP01, cell growth decreased in a dose-dependent manner compared to untreated MCF7 cells. This inhibition of cell growth was significant from day 4 at a concentration of 50 µg/mL and from day 7 at a concentration of 20 µg/mL.

**Figure 2:**
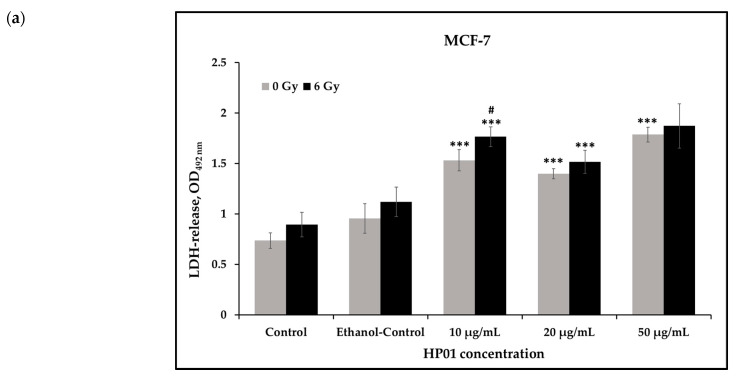

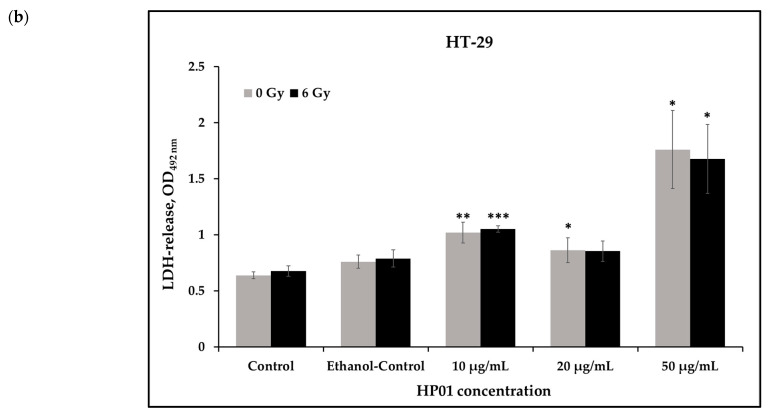
Measurement of cytotoxicity of *Hypericum perforatum* extract was performed 24 h after treatment of (**a**) MCF-7 and (**b**) HT-29 cells via release of lactate dehydrogenase (LDH). Triplicates were scored for each dose. Data from four (MCF7) and three (HT-29) independent experiments are presented as mean values ± SD. Asterisks illustrate significances calculated in relation to controls without HP01 treatment (for non-irradiated experiments: 0 µg/mL HP01; 0 Gy; for irradiated experiments: 0 µg/mL HP01; 6 Gy): * *p* ≤ 0.05, ** *p* ≤ 0.01, *** *p* ≤ 0.001. Hash sign illustrate significance between data from treatment with the same HP01 concentration, among non-irradiated (0 Gy) and irradiated (6 Gy) attempts (^#^ *p* ≤ 0.05).

For HT-29 cells, the findings indicated that low concentrations of HP01 (1 µg/mL, 5 µg/mL, and 10 µg/mL) did not impact the growth of HT-29 cells (Figure 2b). However, growth inhibition began at a concentration of 20 µg/mL HP01, which occurred in a dose-dependent manner. This growth inhibition was statistically significant from day 4 from a concentration of 50 µg/mL and at day 10 from a concentration of 10 µg/mL. Compared to MCF-7 cells, HT-29 cells exhibited lower sensitivity to the HP01 compound.

### 2.2. Toxicity of Hypericum perforatum Extract HP01 on Tumor Cells

To evaluate the toxicity of HP01, both alone and in combination with radiation (6 Gy), a lactate dehydrogenase (LDH) assay was performed (Figure 2).

It was initially observed that MCF-7 cells treated with HP01, without irradiation, consistently exhibited a higher LDH release than control cells (Figure 2a). Starting at a concentration of 10 µg/mL, there was a highly significant increase in LDH release relative to the control. Similarly, after additional irradiation, HP01-treated cells exhibited a significantly elevated LDH release starting from a concentration of 10 µg/mL compared to the untreated cells. Overall, the level of LDH release was higher in MCF-7 cells 24 h post-6 Gy irradiation compared to non-irradiated cells, with a significant increase in LDH release observed in irradiated cells treated with 10 µg/mL HP01 compared to non-irradiated controls.

The observations of cytotoxicity of HP01 on HT-29 cells were similar (Figure 2b). While untreated and solvent control cells exhibited low LDH release, the LDH release in non-irradiated colon carcinoma cells was significantly increased following HP01 treatment starting from a concentration of 10 µg/mL. In 6 Gy-irradiated HT-29 cells, the LDH release at 10 µg/mL was significantly higher compared to the control. Cells treated with 20 µg/mL did not show a significant change in LDH release, whereas a concentration of 50 µg/mL HP01 resulted in a significantly increased LDH release compared to the control.

### 2.3. Influence of Hypericum perforatum Extract HP01 on the Radiosensitivity of Tumor Cells

**Figure 3:**
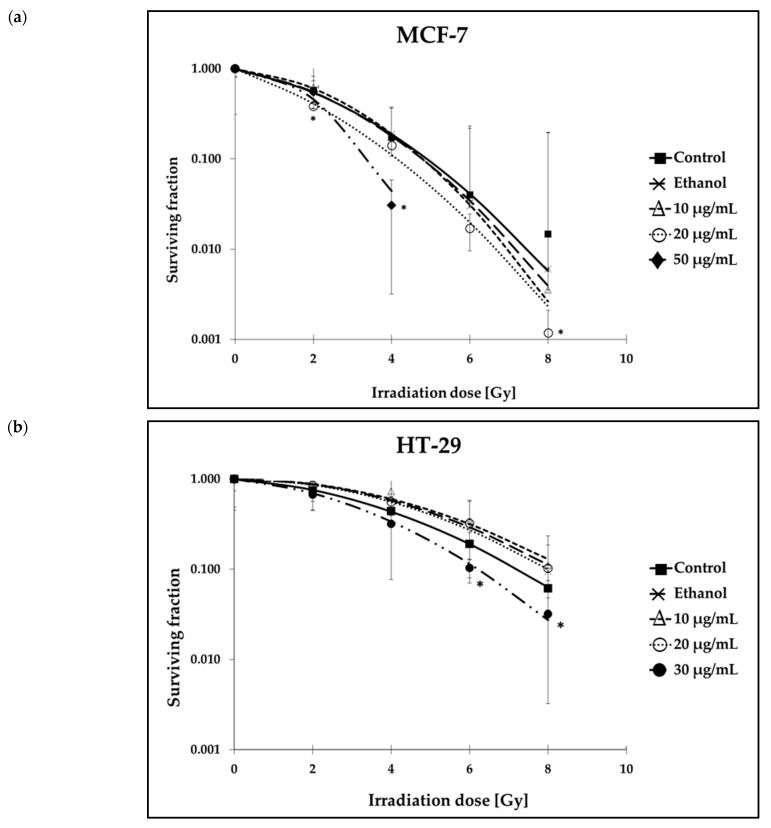
Clonogenic survival of (**a**) MCF-7 and (**b**) HT-29 cells after treatment with *Hypericum perforatum* extract HP01. HP01 treatment was performed 24 h after seeding and 24 h before irradiation treatment of cells. Data from at least three independent experiments are presented as normalized mean values of numbers of colonies ± SD. Asterisks illustrate significances: * *p* ≤ 0.05.

Colony formation assay was performed to investigate the clonogenic survival of carcinoma cells under the influence of HP01 and ionizing radiation (Figure 3).

The long-term survival of MCF-7 cells decreased with increasing radiation doses (Figure 3a), independent of treatment with or without HP01. Without the influence of HP01, the surviving fraction after a radiation dose of 2 Gy (=SF2) was 0.57, decreasing to 0.015 after exposure to 8 Gy. After the addition of HP01 extract, the effect on long-term survival was concentration-dependent, increasing with higher drug concentrations. At a drug concentration of 20 µg/mL HP01, the clonogenic survival of irradiated MCF-7 cells was significantly reduced. A clear radiation-sensitizing effect for MCF-7 cells was demonstrated at higher drug concentration of 50 µg/mL HP01.

The control analysis of HT-29 cells indicated that clonogenic survival of colon carcinoma cells also decreased significantly with increasing irradiation doses (Figure 3b). Without HP01 treatment, the SF2 for these cells was 0.75, which dropped to 0.06 at the maximum radiation dose of 8 Gy, indicating that HT-29 cells were more radiation resistant than MCF-7 cells. With HP01 at concentrations of 10 µg/mL and 20 µg/mL, the survival curves were comparable to the ethanol control curve without the drug. However, a statistically significant reduction in clonogenic cell survival was observed in HT-29 cells at a higher drug concentration of 30 µg/mL HP01 when irradiated with 6 Gy or 8 Gy. Thus, a radiosensitizing effect of HP01 at higher concentrations was demonstrated for both cell lines. In comparison of both cell lines, notably, the synergistic effect of ionizing radiation and HP01 on reducing clonogenic survival was most pronounced in the MCF-7 cells.

### 2.4. Influence of Hypericum perforatum Extract HP01 on Number of DNA Double-Strand Breaks and Their Repair Capacity

**Figure 4:**
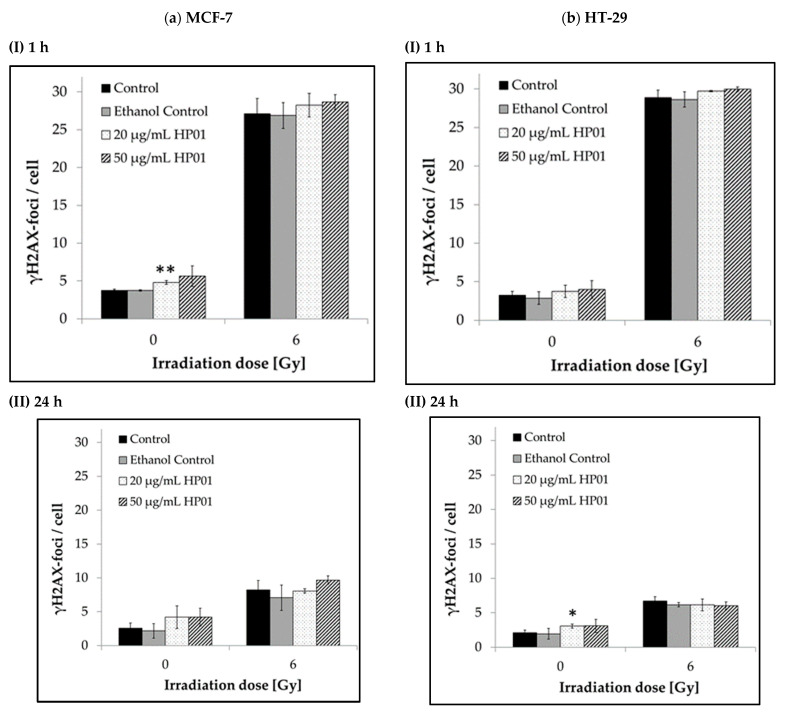
DNA double-strand breaks in cells under different concentrations of *Hypericum perforatum* extract HP01. γH2AX foci were scored in (**a**) MCF-7 and (**b**) HT-29 cells (I) 1 h and (II) 24 h after radiation with 6 Gy or 0 Gy (control) and treatment with different HP01 concentrations (20 µg/mL, 50 µg/mL). Data from three independent experiments are presented as mean values ± SD. Asterisks illustrate significances: * *p* ≤ 0.05, ** *p* ≤ 0.01.

DNA double-strand breaks (DSBs) were quantitatively analyzed in MCF-7 and HT-29 cells following treatment with HP01 and irradiation using the γH2AX assay. The γH2AX foci were assessed both one hour after irradiation and following a repair period of 24 h (Figure 4).

Treating MCF-7 or HT-29 cells with HP01 alone resulted in a significant increase in the number of DNA double-strand breaks (DSBs), as indicated by a higher count of γH2AX foci observed at 0 Gy (Figure 4). In cells treated with 20 µg/mL HP01 and analyzed one hour after sham irradiation, the mean number of DSBs increased compared to the untreated control. For MCF-7 cells, this increase in DSBs was statistically significant (*p* < 0.01). The γH2AX foci counts for both cell lines showed only slight variation between different HP01 concentrations (20 µg/mL, 50 µg/mL). The effect was also evident in the samples analyzed 24 h after sham irradiation.

After exposure to 6 Gy radiation, pronounced time-dependent effects on the γH2AX foci count were observed. For both cell lines, irradiation with 6 Gy drastically increased the number of γH2AX foci after one hour (Figure 4, I). Irradiation of the cells with 6 Gy resulted in a significant increase (*p* < 0.001) in DSBs after one hour, reaching more than 7-fold for MCF-7cells and approximately 9-fold for HT-29 cells compared to the sham-irradiated control cells. MCF-7 cells treated with HP01 showed a more than 5-fold increase in DSBs, while HT-29 cells exhibited more than 8-fold increase one hour after irradiation, both with strong statistical significance. Due to the high number of foci, no significant differences were observed between HP01-treated and non-drug-treated cells.

To evaluate the capacity of cells to repair DNA DSBs, residual foci were assessed after a 24 h repair period. In both HP01-treated cell lines, no increase in the number of residual γH2AX foci was observed 24 h post-radiation compared to unirradiated cells.

### 2.5. Effect of Hypericum perforatum Extract HP01 on Cell Cycle

**Figure 5:**
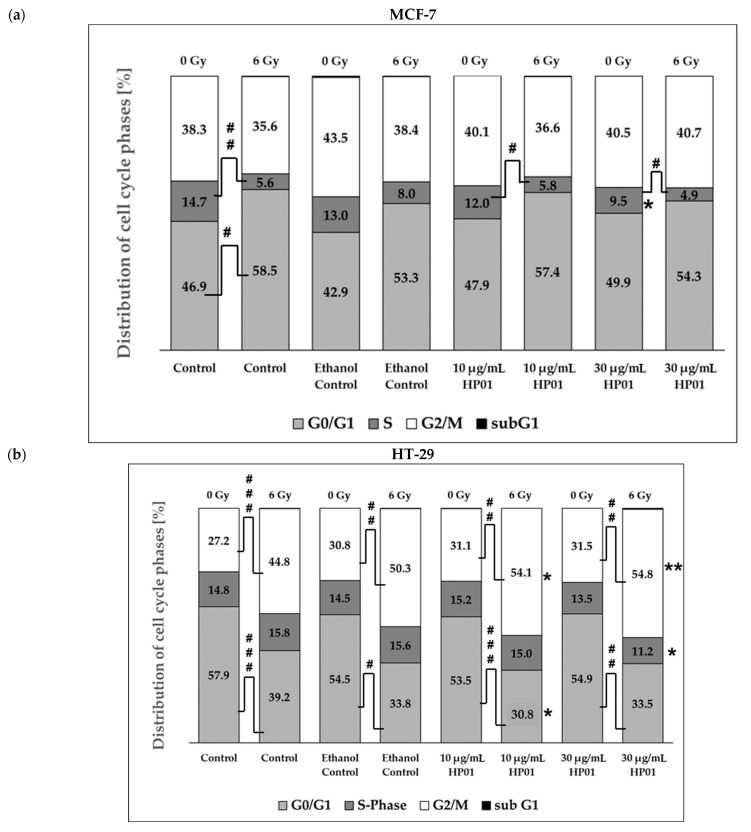
Cell cycle distribution of *Hypericum perforatum* extract HP01-treated MCF-7 (**a**) and HT-29 (**b**) cells in combination with ionizing radiation (6 Gy) or non-irradiation (0 Gy). HP01 was added to the cells 24 h before irradiation. Cells were fixed 24 h after irradiation (48 h HP01 treatment). The proportion of sub-G1-phase cells was less than 0.5% in each case. For the three independent experiments significances were calculated in relation to controls without HP01 treatment (for non-irradiated experiments: 0 µg/mL HP01; 0 Gy; for irradiated experiments: 0 µg/mL HP01; 6 Gy) and illustrated by asterisks (* *p* ≤ 0.05, ** *p* ≤ 0.01). Hash signs illustrate significance between data from treatment with the same HP01 concentration among non-irradiated (0 Gy) and irradiated (6 Gy) attempts (^#^ *p* ≤ 0.05, ^##^ *p* ≤ 0.01, ^###^ *p* ≤ 0.001).

The aim was to determine whether HP01 influences the cell cycle of MCF-7 or HT-29 cells. To achieve this, cells were treated with 10 µg/mL or 30 µg/mL HP01 and analyzed 48 h later using flow cytometry (Figure 5).

During the analysis of cell cycle distribution, it was observed that, while the addition of 10 µg/mL HP01 did not affect the MCF-7 cells, treatment with 30 µg/mL HP01 caused the tumor cells to arrest in the G0/G1 phase, concurrently leading to a decrease in the proportion of S-phase cells. In contrast, the effect observed in the colon carcinoma cells (HT-29) was different. Here, the addition of 30 µg/mL HP01 led to an increase in the proportion of tumor cells arrested in the G2/M phase, while the proportion of cells in the G0/G1 phase decreased. This effect cell line already evident at the lower concentration of 10 µg/mL.

Subsequently, the cells were irradiated with 6 Gy 24 h after drug addition. While the treatment with HP01 alone produced qualitatively different effects on the two cell lines, no differences were detected when irradiation was combined with the drug. Compared to the irradiated controls without the drug, both MCF-7 and HT-29 cells arrested in the G2/M phase upon addition of 30 µg/mL HP01, accompanied by a simultaneous reduction in the S phase and the G0/G1 phase. This effect was notably pronounced in the HT-29 cells at 10 µg/mL HP01 concentration, whereas in MCF-7 cells, only a tendency was observed at this lower concentration, with a clear effect manifesting only at the higher dose of HP01 (30 µg/mL).

## 3. Discussion

The aim of this study was to investigate the effects of *Hypericum perforatum* extract HP01, both alone and in combination with ionizing radiation, on the human tumor cell lines MCF-7 (breast carcinoma) and HT-29 (colon carcinoma).

Based on the data of our study, dose- and time-dependent growth inhibition was demonstrated for both cell lines, with the MCF-7 cell line appearing to be slightly more sensitive. *Hypericum*-mediated inhibition of cell growth has already been demonstrated in other human cell types and carcinoma cell lines, such as hepatocellular carcinoma, colorectal cancer, and non-small cell lung carcinoma [25,26,27,28,29]. In all studies where growth inhibition was examined in more detail, apoptosis-mediated inhibition was found [25,26,27,28]. Therefore, in our study, we also assume that the observed growth inhibition by *Hypericum* was apoptosis-mediated.

However, there is ample literature not only on *Hypericum* itself, but also on its components. For example, hyperforin, a bioactive compound of *Hypericum*, has demonstrated growth inhibition in various breast cancer cells, as well as in cells of squamous cell carcinoma, malignant melanoma, and lymphoma [30]. Hyperoside, another bioactive compound of *Hypericum*, also exhibits growth-inhibiting and apoptosis-inducing properties in A549 human non-small cell lung cancer cells [18]. On the other hand, hypericin alone show only minor proliferation inhibition and apoptosis induction compared to the full-strength *Hypericum* extract [16,29]. Various colon carcinoma cell lines have responded to treatment with skyrin, a secondary metabolite of *Hypericum,* resulting in reduced metabolic activity and growth inhibition [31]. Researchers in multiple studies have concluded that the growth-inhibitory and cytotoxic effects of *Hypericum* are induced by apoptosis. The apoptosis-inducing effect of *Hypericum* has been frequently documented in the literature, demonstrating that annexin V and dead cell markers increase in a dose-dependent manner due to St. John’s wort herb in MCF-7 cells [29]. Similar results have been reported in studies involving different tumor entities, with a dose-dependent induction of cell apoptosis in human HepG2 hepatoma cells using an ethyl acetate extract of *Hypericum japonicum* [28]. Furthermore, a significant increase in apoptosis was observed in HCT-116 colon carcinoma cells after treatment with *Hypericum triquetrifolium* extract [17]. The intrinsic pathway of apoptosis appears to play a primary role in this class of substances, where mitochondria-dependent apoptosis leads to the release of proapoptotic molecules such as cytochrome c and apoptosis-inducing factor (AIF). In non-small-cell lung cancer (NSCLC) cells A549, the expression levels of mitochondrial cytochrome c and AIF increased following the addition of hyperoside [10]. Additionally, increased activities of caspase 3 and 9 were noted in the cells, resulting in programmed cell death.

The cytotoxic effect of *Hypericum* described in the cited studies was also a focal point of our investigation. We employed the detection of lactate dehydrogenase (LDH) release, an important indicator of the toxicity of a substance on (tumor) cells. Both MCF-7 and HT-29 cells, with or without irradiation, demonstrated a significant increase in LDH release starting from an HP01 concentration of 10 µg/mL compared to control lines. In the literature, the treatment of two colon carcinoma cell lines (CO-115 and HCT-15) with extracts from two *Hypericum* species (*H. perforatum* and *H. androsaemum*) for 48 h has shown that the extracts are only toxic to the cells at concentrations of 200 µg/mL and above [27]. Another study on HCT-116 colon carcinoma cells treated with *Hypericum triquetrifolium* extract for 24 h indicated that only concentrations from 500 µg/mL and higher are toxic, as evidenced by a significant increase in LDH release [17]. In contrast, our study observed a significantly increased LDH release and, thus, proven cytotoxicity from a relatively low concentration of 10 µg/mL *H. perforatum* extract, which may be related to the specific metabolite profile of the genotype (HP01) used in our study, containing a relatively high total naphthodianthrone content of approximately 9.7 mg/g. Further tests with extracts containing lower amounts of biologically active hypericin are necessary to determine the extent to which the composition of the extract contributes to the degree of cytotoxicity.

To assess the impact of *Hypericum* on the cellular response to radiation, clonogenic survival assays were performed using clinically relevant doses ranging from 2 Gy to 8 Gy. A survival fraction can be determined from the cell survival curves obtained from the results of the colony formation assay, which serves as a measure of the radiation sensitivity of cells. This sensitivity can be influenced by certain agents. For example, some substances can have a radioprotective effect, promoting the clonogenic survival of cells and thus protecting them against the toxic effects of ionizing radiation [32]. Conversely, other substances can have a radiosensitizing effect, thereby enhancing the efficacy of radiotherapy [33,34,35].

In our study without the drug, exposure to increasing radiation doses resulted in a decrease in the survival of both cell lines. The dose–response relationship for treatment with ionizing radiation was demonstrated in both MCF-7 and HT-29 cells. The MCF-7 cells showed a significant decrease in clonogenic cell survival with increasing radiation doses compared to the control without HP01. At the maximum irradiation dose of 8 Gy, the survival fraction was only 0.015. The high radiation sensitivity of MCF-7 cells aligns with the literature. El-Awady et al. [36] verified the high radiosensitivity of MCF-7 cell lines in their studies on the radiosensitivity of various human tumor cell lines. In contrast, the HT-29 colon carcinoma cells exhibited lower sensitivity to ionizing radiation compared to the MCF-7 cells, a finding consistent with previous studies [37,38]. It is known that ionizing radiation can lead to a loss of DNA integrity in tumor cells, resulting in clonogenic cell death. Research has shown that this is due to an arrest in cell cycle at the transition from G2 phase to the M phase. This phenomenon, known as the G2/M block, is a well-documented biological response to ionizing radiation [39]. If the cell is unable to repair the DNA damage during this blockade, apoptosis is initiated.

The treatment of the tumor cells with a combination of irradiation and HP01 in higher concentrations resulted in a radiosensitizing effect in both cell lines; the decrease in clonogenic cell survival with increasing radiation doses was enhanced. The MCF-7 cells displayed this effect at a lower *Hypericum* concentration (from 20 µg/mL) compared to the HT-29 cells (from 30 µg/mL), indicating that they are, therefore, more sensitive. To our knowledge, this is the first in vitro study that describes a radiosensitizing effect of a *Hypericum* extract. As such, a detailed comparison with the literature on a radiosensitizing effect of *Hypericum perforatum* in vitro is not possible at this time. As previously mentioned, there are several active substances known to cause radiosensitizing effects, which are routinely used in clinics for patients undergoing radiochemotherapy. Depending on the tumor type, various cytostatic agents, such as taxanes, cisplatin, temozolomide, and 5-fluorouracil, or their combinations, demonstrate remarkable supra-additive effects in reducing tumor size [6,7,8]. There is a pressing need to identify natural radiosensitizing substances with fewer severe side effects compared to chemical cytostatic agents. This study highlights *Hypericum perforatum* extract HP01 as a promising candidate for a natural radiosensitizer, but further investigations (including in vivo and clinical studies) must be conducted to determine whether the use of this promising substance in the clinic is feasible in the future.

The differing sensitivity of the two cell lines to the drug and radiation may have various causes. It is well known that differing expression levels of the pro-apoptotic gene p53 can play a significant role in this context. The HT-29 cells used in this study are characterized by a mutation in the p53 gene [40], while the MCF-7 cells express the wild-type p53 gene [41]. Additionally, the expression of other genes, such as EGFR, CDK2, BRAF, BCL2, etc., may also influence sensitivity.

In order to further characterize the radiosensitizing effect of the *Hypericum* extract, initial studies on the mechanism of action were conducted. The induction of DNA double-strand breaks (DSBs) is a key radiobiological effect of ionizing radiation. This study examined sublethal radiation damage, which may be repairable to ensure the survival of clonogenic cells. Therefore, the impact of HP01 on the quantity of DNA DSBs and their repair capacity was assessed using the γH2AX assay. The results showed that HP01 increased the number of γH2AX foci, indicating an increase in DNA DSBs, independently of irradiation. However, the substance had no effect on the repair capacity of radiation induced DNA DSBs. Thus, 24 h after irradiation, the presence of HP01 did not change the number of γH2AX foci compared to the irradiated control groups lacking the drug. This finding applied to both the MCF-7 and HT-29 cell lines. Other studies have shown that *Hypericum adenotrichum* extract could cause DNA DSBs in HL-60 leukemia cells, potentially leading to instability of genomic DNA [42]. However, based on the results of our study, it can be concluded that the radiosensitizing effect of HP01 is not caused by residual DSB.

The analysis of the cell cycle using flow cytometry aimed to further elucidate the causal mechanisms underlying the results from growth curves and colony formation assays. This study revealed a radiation-induced G2/M arrest in HT-29 cells, as documented in the literature for various tumor cell lines [43,44]. In contrast, the MCF-7 cell line displayed a G0/G1 arrest based on cell cycle analysis. Both the restriction point of the G2/M phase and the transition from the G1 to the S phase are characteristic of cell cycle arrest following DNA damage and may subsequently lead to the initiation of apoptosis [45]. The addition of HP01 induced a G2/M arrest in both the HT-29 and MCF-7 cell lines, thereby enhancing the effect of ionizing radiation. Research in the field of radiobiology has established that cellular radiosensitivity is influenced by the cell cycle phase at the time of irradiation, with mammalian cells being most sensitive to radiation during the G2/M phase [46]. If HP01 causes a greater accumulation of cells in G2/M arrest, this suggests that the bioactive substance increases the cells’ sensitivity to radiation. This observation can explain the increased radiation response noted in the colony formation assay and the resulting reduced cell survival. The literature widely acknowledges that the accumulation of cells in the G2/M phase can be a primary mechanism underlying the radiosensitizing effects of various substances, as demonstrated for taxanes and epothilones [47].

In summary, this study has demonstrated the cytotoxic and radiosensitizing effects of *Hypericum perforatum* extract HP01. The substance significantly enhances radiation effects in tumor cells. This effect is not due to an influence on DNA DSB repair, but rather to the accumulation of cells in the G2/M phase of the cell cycle. These findings provide compelling evidence of the high potential for HP01 to be used in combined radio-chemotherapy in the future. It should be noted that these results originate from preclinical experimental systems, and while specific radiosensitization can ultimately only be unequivocally proven in vitro, translating experimental data (via animal models) into clinical practice is often not straightforward. Further studies are needed to gain deeper insights into the mechanism of the radiosensitizing effect of HP01.

## 4. Materials and Methods

### 4.1. Materials

Dulbecco’s modified Eagle’s medium (DMEM) containing high glucose (4.5 g/L), and L-Glutamine, as well as sodium pyruvate were purchased from Capricorn Scientific (Ebsdorfergrund, Germany), and phosphate-buffered saline (PBS) was received from PAN Biotech GmbH (Aidenbach, Germany). Fetal bovine serum (FBS) and Trypsin (0.05%)/EDTA (0.02%) were obtained from PAN Biotech GmbH (Aidenbach, Germany) and penicillin/streptomycin (P/S) from Sigma-Aldrich (Merck KGaA, Darmstadt, Germany). HP01 dry extract was provided from Institute for Breeding Research on Horticultural Crops, Julius-Kuehn-Institute—Federal Research Centre for Cultivated Plants (Quedlinburg, Germany).

#### 4.1.1. Production of *Hypericum perforatum* Dry Extract (HP01)

Plant material was produced as described before [48]. In brief, seeds of *Hypericum perforatum* were obtained from the Julius Kühn Institute (Quedlinburg, Germany). Following 14 days of germination at 18 °C in a climate chamber, seedlings were cultivated in a greenhouse for 12 weeks at 22 °C under halogen vapor lamp illumination with a 10 h short-day photoperiod. Mature plants were transplanted into open-field conditions. Inflorescences were harvested and dried at 30 °C for three days, and a composite sample was prepared.

Extraction was performed as previously described [48]. The plant material was ground in accordance with the European Pharmacopoeia (Ph. Eur. 10.0, 1438 (12/2020)). For the extraction process, 200 g of ground *Hyperici herba* underwent ultrasonic-assisted extraction using 1000 mL of 70% (*v*/*v*) ethanol at 50 °C for 40 min. After collecting the solvent, a second extraction was performed with 500 mL of fresh solvent to ensure exhaustive extraction. The combined solvent fractions were concentrated and dried to completeness using vacuum rotary evaporation, yielding the dry extract HP01.

#### 4.1.2. Composition of *Hypericum perforatum* Dry Extract (HP01)

##### HPLC Analysis

The HPLC analysis for hypericin was performed using a C18 column (Agilent, Pursuit XRs C18, 100 Å, 4.6 × 150 mm, 3 µm, Agilent Technologies, Santa Clara, CA, USA). The column temperature was maintained at 40 °C using a column oven (VWR Hitachi 5310, VWR International, Radnor, PA, USA). The mobile phase consisted of 39% ethyl acetate (R), 41% sodium dihydrogen phosphate solution (15.6 g/L) adjusted to pH 2 with 85% phosphoric acid (R), and 160% methanol (R). The flow rate was set at 1.0 mL/min using an HPLC pump (VWR Chromaster pump 5160, VWR International, Radnor, PA, USA), and detection was carried out using a spectrometer at 590 nm and/or a fluorescence detector (Ex = 470 nm, Em = 590 nm). A sample volume of 10 µL was injected using an autosampler (VWR Hitachi 5260, VWR International, Radnor, PA, USA), and the chromatographic run time was 15 min.

For hyperforin and flavonoids, the same column was used, and the temperature was maintained at 25 °C. The mobile phase included Mobile Phase A (85% phosphoric acid and water in a ratio of 3:1000 *v*/*v*) and Mobile Phase B (85% phosphoric acid and acetonitrile in a ratio of 3:1000 *v*/*v*). The gradient program began with 82% A and 18% B, transitioning to 47% A and 53% B over 18 min, then to 3% A and 97% B for the remainder of the run. The flow rate varied from 0.8 mL/min to 1.2 mL/min during the run. Detection was performed at 360 nm for flavonoids and 275 nm for hyperforin and adhyperforin. A sample volume of 10 µL was injected, and the chromatographic run time was 35 min.

The composition consisted of key compounds: rutin at 1.978 mg/g, total flavonoids at 9.001 mg/g, hyperforin at 3.215 mg/g, adhyperforin at 0.299 mg/g, and hypericin at 0.064 mg/g. Total flavonoids were determined as described in European Pharmacopeia (Ph. Eur. 10.0, 1438 (12/2020)).

##### Photometric Analysis of Naphtodianthrones

At 588 nm, both hypericin and pseudohypericin exhibited absorption, making photometric measurement of the total extract a suitable approach to quantify the total naphthodian-throne content. For this purpose, calibration was performed using a hypericin standard on the SpectraMax^®^ Plus 384 system (Molecular Devices, San Jose, CA, USA). Subsequently, 5 µL of the 100 mg/mL stock solution of HP01 was measured in two technical replicates against an ethanol blank. The resulting total amount of soluble naphthodianthrones was determined to be 9.7 mg/g.

### 4.2. Methods

#### 4.2.1. Preparation of *Hypericum perforatum* Extract HP01 Solution for Cell Treatment

To prepare the HP01 stock solution for cell culture, 3.5 g of HP01 dry extract was dissolved in 35 mL of 70% ethanol to achieve a concentration of 100 mg/mL. The solution was homogenized using an ultrasonic bath, followed by vortexing. Dilution solutions of various concentrations were prepared under sterile conditions. For a concentration of 50 mg/mL, the stock solution was diluted 1:2 with 70% ethanol (Dilution Solution 1, DS1). For a concentration of 10 mg/mL, the stock solution was diluted 1:10 with 70% ethanol (DS2). For a concentration of 1 mg/mL, the 10 mg/mL solution (DS2) was diluted 1:10 with 70% ethanol (DS3).

#### 4.2.2. Cell Lines

Human breast epithelial cells (MCF-7; American Type Culture Collection; Wesel, Germany, catalog no.: HTB-22^TM^) and the colon adenocarcinoma cell line (HT-29, DSMZ, Braunschweig, Germany; catalog no.: ACC 299) were cultivated at 37 °C with 5% CO_2_ in DMEM, supplemented with 10% FBS and 1% penicillin/streptomycin. To maintain exponential growth, the cell lines were passaged twice weekly.

#### 4.2.3. Growth Curves

Cells were cultured for 24 h at 37 °C and 5% CO_2_, with the seeding day designated as t = 0 h. The analysis was conducted in 24-well plates with 5 × 10^3^ cells per well in 1 mL medium. The HP01 solution was used in cell culture with increasing concentrations of 0 µg/mL (control), 1 µg/mL, 5 µg/mL, 10 µg/mL, 20 µg/mL, 50 µg/mL, and 100 µg/mL. In addition, the solvent was checked by adding 500 µg/mL ethanol (70%). Cell counts were determined at 24 h post-seeding and every 72 h thereafter in triplicate. For that, cells were trypsinized and the cell number was counted by a Casy^®^ Counter (Omni Life Science GmbH &Co. KG, Bremen, Germany). The mean cell counts from triplicate measurements were used to characterize cell growth kinetics for one experiment. At least three independent experiments were performed.

#### 4.2.4. Irradiation

The cells were exposed to radiation at room temperature, either in cell culture flasks or multi-well plates, using a Versa HD linear accelerator manufactured by Elekta Ltd. (Crawley, UK). The procedure employed a photon energy of 6 MV and a dose rate of 6.6 ± 0.6 Gy/min, with single doses of 2 Gy, 4 Gy, 6 Gy, or 8 Gy. To account for environmental conditions, a control sample (0 Gy) was also brought into the irradiation room for comparison.

#### 4.2.5. LDH Assay

The lactate dehydrogenase (LDH) assay is an in vitro cytotoxicity test that photometrically detects the enzymatic activity of LDH. This enzyme is typically localized in the cytoplasm of cells and is released into the culture medium upon cell damage or lysis, where it can be quantified. For this assay, 2.5 × 10^4^ cells (MCF-7 or HT-29) were seeded in 96-well plates. After 24 h of incubation at 37 °C and 5% CO_2_, the cells were treated with 10 µg/mL, 20 µg/mL, and 50 µg/mL HP01. Following an additional 24 h of incubation at 37 °C and 5% CO_2_, the cells were irradiated with 6 Gy. The control group was sham-irradiated. After another 24 h of incubation at standard conditions, the cell culture supernatant was collected and the LDH assay was performed according to the manufacturer’s instructions (LDH Cytotoxicity Detection Kit, Sigma-Aldrich, Merck KGaA, Darmstadt, Germany). Absorbance was measured photometrically at a wavelength of 492 nm at least in triplicate per experiment, and at least three independent experiments were performed.

#### 4.2.6. Colony Forming Assay

Two days prior to irradiation, 1 × 10^3^ cells from the MCF-7 and HT-29 cell lines were seeded in duplicate into 25 cm^2^ culture flasks, corresponding to each dose. After 24 h, the HP01 compound was administered in different concentrations (10 µg/mL, 20 µg/mL, 30 µg/mL, 50 µg/mL). A control without HP01 as well as a solvent control with ethanol were carried out. The cells were then irradiated at doses of 0 Gy (control), 2 Gy, 4 Gy, 6 Gy, and 8 Gy. Seven days post-irradiation, the medium was replaced with fresh drug-free complete medium. After an additional seven days, the colonies were fixed with 70% ethanol for 10 min, followed by staining with a 1% crystal violet solution for 5 to 10 min. Only colonies with at least 50 cells were considered for the evaluation, and these were counted under the microscope. Afterwards, the survival fraction (SF) was calculated.

#### 4.2.7. γH2AX Assay

The DNA double-strand breaks (DSBs) were detected by immunofluorescence of γH2AX foci. Chambered coverslips with four chambers (Nunc^TM^ LabTek^TM^, Rochester, NY, USA) were used for the assay. Twenty-four hours after seeding the cells, HP01 was added (10 μg/mL or 50 μg/mL) [35]. After an additional 24 h of incubation, the cells were irradiated with 6 Gy using a linear accelerator. Fixation was performed either 1 h or 24 h post-irradiation. Since the maximum of DNA double-strand breaks (DSBs) occurs approximately 30–90 min after damage, the first fixation time point was set at 1 h. The 24 h time point was selected to assess the completion of radiation-induced DNA-DSB repair, providing insights into the cells’ repair capacity. Detached slides were fixed and stained following a previously established protocol [35]. The foci in 50 cell nuclei were counted per experiment using fluorescence microscopy. Each experiment was performed in duplicate, with the average of the duplicate counts representing the result of a single experiment. In total, three independent experiments were conducted.

#### 4.2.8. Cell Cycle Analyses

Cells were seeded at an appropriate density and replaced with serum-free medium 24 h after seeding to initiate cell cycle synchronization. A further 24 h later, HP01 was added at concentrations of 10 µg/mL and 30 µg/mL. Irradiation was performed 24 h after the addition of HP01 at doses of 6 Gy or 0 Gy (control) and was performed in at least three independent experiments for each experimental approach. Twenty-four hours after irradiation, the cells were fixed and permeabilized overnight in 70% ethanol (*v*/*v*) at −20 °C. Subsequently, the cells were stained with propidium iodide at a concentration of 50 μg/mL. The samples were measured using the CytoFLEX flow cytometer (Beckman Coulter, Krefeld, Germany). Data analysis was performed using CytExpert for Windows, version 2.6 (Beckman Coulter, Krefeld, Germany) [49].

#### 4.2.9. Statistical Methods

Calculations were based on the averages derived from a minimum of three independent experiments. For all experiments, normality was assessed using the Anderson–Darling test. Significant differences were determined using the two-tailed Student’s *t*-test for independent samples for parametric data and the Mann–Whitney U Test for non-parametric data. The following significance levels were established: * *p* ≤ 0.05, ** *p* ≤ 0.01, *** *p* ≤ 0.001.

## Data Availability

The data presented in this study are available on request from the corresponding author.
